# A Cognitive Neuropsychological and Psychophysiological Investigation of a Patient Who Exhibited an Acute Exacerbated Behavioural Response during Innocuous Somatosensory Stimulation and Movement

**DOI:** 10.1155/2004/458327

**Published:** 2004-06-09

**Authors:** N. M. J. Edelstyn, S. R. Baker, S. J. Ellis, P. Jenkinson

**Affiliations:** ^1^Department of PsychologyUniversity of KeeleStaffordshireUK; ^2^Department of NeurologyRoyal InfirmaryStoke-on-TrentStaffordshireUK

## Abstract

We report findings from a cognitive neuropsychological and psychophysiological investigation of a patient who displayed an exacerbated acute emotional expression during movement, innocuous, and aversive somatosensory stimulation. The condition developed in the context of non-specific white matter ischaemia along with abnormalities in the cortical white matter of the left anterior parietal lobe, and subcortical white matter of the left Sylvian cortex.

Cognitive neuropsychological assessment revealed a pronounced deficiency in executive function, relative to IQ, memory, attention, language and visual processing. Compared to a normal control group, the patient [EQ] displayed a significantly elevated skin conductance level during both innocuous and aversive somatosensory stimulation. His pain tolerance was also significantly reduced. Despite this, EQ remained able to accurately describe the form of stimulation taking place, and to rate the levels of pain intensity and pain affect.

These results suggest that EQ’s exaggerated behavioural response and reduced pain tolerance to somatosensory stimulation may be linked to cognitive changes, possibly related to increased apprehension and fear, rather than altered pain intensity or pain affect per se.

## 1. Introduction

This study investigates the relationship between arousal, as reflected in skin conductance level (SCL), pain tolerance, and subjective ratings of pain intensity and pain affect in a patient who displayed an exacerbated acute emotional expression during movement, innocuous and aversive somatosensory stimulation. The patient's (EQ) response to innocuous somatosensory stimulation initially suggested that he may be suffering from allodynia, a condition characterised by a 'misinterpretation' of somatosensory information which evokes a painful experience for intensities clearly below the normal pain threshold (e.g. [19]). However, EQ differed from previously reported allodynic patients (e.g. [4,19,20]) on a number of key points. Firstly, a pain response could be elicited during mild stimulation or movement of any part of EQ's body, whereas allodynic pain is typically lateralised to a small contralesional area of the body . Secondly, allodynic stimulation typically results in a 'normal' pain response which encompasses both the sensory-discriminative [i.e. the spatial, temporal and intensity properties of the stimulus] as well as the affective-motivational dimension

ISSN 0953-4180/04/$17.00 © 2004 – IOS Press and the authors. All rights reserved

(i.e. the unpleasantness of the stimulus as well as the behavioural and autonomic reactions) (e.g. [12,19]). However, EQ's acute vocalised pain response lacked an affective/ motivation dimension – the tone of his voice was neutral and he didn't grimace. Furthermore, once somatosensory stimulation ceased, EQ was able to accurately describe the form of activity which had just taken place e.g. stroking his hand.

A number of earlier studies have reported altered experiences of innocuous and noxious stimuli in patients following frontal leucotomy (e.g. [3,10]), prefrontal lesions [9,21], bilateral anterior capsulotomy [25] combined with bilateral cingulotomy [6]. For example, both Chapman et al. and Hardy reported exaggerated reactions (e.g. wincing) and withdrawal responses to mildly painful stimuli such as pin prick, in the presence of unchanged or slightly elevated pain threshold levels. More recently, Talbot et al. and Davis et al. found a decrease in pain intensity and pain affect following surgery, although pain tolerance for cold stimuli was also decreased. Both Talbot and Davis suggested that blocking input to the anterior cingulate and prefrontal cortices reduced both the perceived intensity and unpleasantness of noxious stimuli. Whereas the reduction in pain tolerance was attributed to a disinhibition in cortical control of spinal reflexes.

The aim of this case study was to determine whether EQ's exaggerated pain response was explicable in terms of an altered perception of noxious and innocuous stimulation rather than a 'misinterpretation' of somatosensory information which evokes a painful experience for intensities clearly below the normal pain threshold.

## 2. Patient report

### 2.1. General background

EQ is a 57 year-old, right handed, former self-employed man, married with two grown up children. He had a cardiac arrest in his home after jogging aged 55. Cardiopulmonary resuscitation was initiated by paramedics approximately five minutes after the arrest. He remained in the intensive care unit for 8 days, and in hospital a further 6 months. He doesn't smoke or drink alcohol, and there was no family history of heart problems.

Prior to his cardiac arrest, he was a healthy, active man, who enjoyed a variety of leisure activities. He was described by his wife as an interested father and affectionate husband. However, since his cardiac arrest, he has lost interest in his former hobbies, his wife and family. In addition, he has become very lethargic and his behaviour is disinhibited. Of particular interest are EQ's very loud claims of pain, which accompany mild somatosensory stimulation and movement of any part of his body. Activities which involve his wife doing things for him, for example washing him in the shower, cutting his nails or putting his shoes on, are associated with extremely high levels of reported pain. EQ becomes 'pain-free' when he is sitting undisturbed, eating, drinking, completing a crossword or jigsaw puzzle.

### 2.2. Neurological examination

Twelve months after his cardiac arrest, when EQ was aged 56, his score on the mini-mental state examination [8] was deficient 15/30. He was disorientated for time but not place. He also was unable to recall three objects after 3 minutes.

Examination of cranial nerves revealed normal functioning. No weakness or increased tone was evident in his upper limbs, although he had some upper motor neurone problems in his right leg with an extensor plantar response on that side. He needed assistance maintaining his balance and walking. The deep tendon reflexes were all present. The Romberg test was positive. Sensation in the limbs revealed that light touch, vibration and proprioception were intact. However, whenever his skin was stimulated with a neurotip [pin prick] he would cry out in pain, but when asked what he felt he would reply "a slight pin prick". Light touch on the legs would also cause him to cry out in pain. There was no evidence of pain asymbolia.

### 2.3. Neuroimaging report

An MRI-scan revealed a prominence of the cortical sulci, cerebral folia and ventricles. There was non-specific white matter ischaemia, with abnormal high T2 signal in the subcortical white matter of the left Sylvian cortex and an additional area of high T2 signal deep in the white matter of the left anterior parietal cortex. No additional abnormalities were identified (see Fig. 1). He was diagnosed with anoxic encephalopathy.

The patient's initials have been changed to preserve anonymity and informed consent was obtained [his wife also provided assent]. This study had local research ethics committee approval, and was performed over a 12 month period.

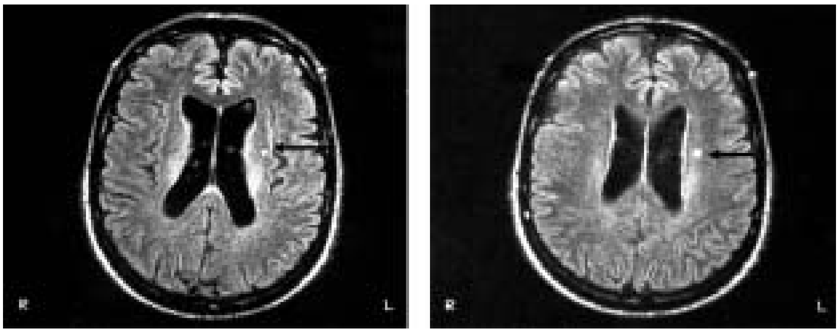

Fig. 1. Magnetic resonance imaging scans showing non-specific white matter ischaemia along with abnormalities in the cortical white matter of the left anterior parietal lobe in plate 1 and subcortical white matter of the left Sylvian cortex in plate 2.

### 2.4. Cognitive neuropsychological assessment

EQ's cognitive neuropsychological profile is presented in Table 1.

His current IQ (*Wechsler Adult Intelligence Scales-Revised* [WAIS-R] [26]) placed him in the borderline range. This score indicated a decline in general intellectual functioning from premorbid levels which placed him in the average range [18]. He was particularly poor on three WAIS-R subtests: *Similarities*, *Object Assembly* and *Digit Symbol*. Successful completion of *Object Assembly* requires orderliness and planning, and is notable in that it does not lend itself to verbalization [16]. Both *Similarities* and *Digit Symbol* are non-specific indicators of brain dysfunction. *Similarities* is particularly vulnerable to disruption of verbal functions, whereas *Digit Symbol* involves the integration of many different components, which include visuomotor processing and visual search [16].

EQ's executive functions were profoundly impaired. He failure to complete a single sorting category on the *The Wisconsin Card Sorting Test* [11], reflected an inability to develop and maintain any form of strategic planning, organised searching, ultilizing feedback to shift cognitive sets, modulating impulsive responding and directing behaviour toward achieving a goal. An inability to modulate impulsive responding was again evident on the *Stroop* test [24] where he was unable to suppress the urge to read the printed word in favour of naming the ink colour.

Abnormalities were also evident on three tests of attention. His performance [measured in terms of median response times] became progressively more impaired as the attentional load was increased from tests of phasic alertness and visual attention to divided attention [28].

EQ displayed a wide range of memory impairments, which included autobiographical (Autobiographical Memory Interview [15]), semantic [WAIS-R Information and Vocabulary subtests], verbal working and short-term [WAIS-R Digit Span and Arithmetic subtests] as well as visual short and long-term memory systems (*Rey Complex Figure Test* [17]).

No abnormalities were detected during assessments of unfamiliar face processing (*Unfamiliar Face Matching Test* [1]), familiar face recognition (sorting famous from unfamiliar faces, and famous face naming) visual object recognition (Minimal Feature and Foreshortened subtests, Birmingham Object Recognition Battery [23]) and language processing (Psycholinguistic Assessments of Language Processing in Aphasia [14]).

## 3. Psychophysiological study

EQ's performance was compared with four age-(mean 53 years, range: 44–56) and gender -matched healthy controls. The healthy volunteers were right handed, physically fit, English was their first language and had no reported history of heart problems, dyslexia, neurological or psychological disturbance. Informed consent was obtained.

### 3.1. Measurement

Skin conductance level (SCL) was recorded continuously using a BIOPAC Remote Monitoring System. SCL was measured using silver/silver chloride electrodes filled with 0.05 M of electrode gel and attached to the distal phalanx of the index and second fingers of the relevant hand [dependent on condition]. Output from the electrodes was recorded on an IBM-compatible PC using BIOPAC systems software.

On arrival at the laboratory (light and temperature controlled: 20.5–21 °C), each participant was provided with instructions about the study and physiological monitoring, and the electrodes were then attached.

### 3.2. Experimental conditions

Following a 10-minute resting period when pre-experimental baseline data was collected, the five conditions were administered. Innocuous somatosensory stimulation consisted of stroking the skin with a tissue. Two aversive conditions consisted of (i) a mild pin prick [using a neurotip] and (ii) a cold water pressor test. Finally, two movement conditions involved active and passive movement, which were participant and examiner initiated, respectively.

In order to determine whether there was an effect of laterality (contingent on the structural MRI evidence of focal pathology in the left hemisphere), the innocuous and aversive (pin prick using a neurotip) stimulation and both movement conditions were performed on each foot separately, and SCRs were recorded from the index and second fingers of the ipsilateral hand.

EQ was told what was going to happen 20 seconds before each condition commenced. The stimulation/movement was then administered, also, for a period of 20 seconds. Cessation of each condition was followed immediately by a 1-minute post-condition recovery period, with the exception of the aversive condition which was followed by a three-minute recovery period.

The cold pressor test involved immersion of the right hand into cold water (3°C) for a maximum period of 5 minutes. Participants were instructed to remove their hand as soon as it became intolerable. Hand immersion was preceded by a 20 second anticipatory period, and followed by a 5 minute recovery period. The patient's clinician (SE) was present throughout the research session.

The same procedure was followed for each of the normal control participants (NC).

### 3.3. Data reduction and analysis

#### 3.3.1. Anticipatory SCL

Anticipatory SCL was averaged over the 10 second period immediately prior to administration of each of the four lateralised conditions and the cold pressor test. Difference scores were calculated between SCL in each of the anticipatory periods relative to the last 60 seconds of the pre-experimental baseline.

#### 3.3.2. Task activity SCL

Task activity SCL was averaged over 20 second periods during administration of each of the four lateralised conditions and the cold pressor test, and over 60-seconds for the post-condition recovery periods. Raw task activity SCL were calculated for each of the ipsilateral conditions. Difference scores were also calculated between task activity SCL in each of the condition periods relative to the last 60 seconds of the pre-experimental baseline.

#### 3.3.3. Qualitative evaluation of evoked sensations

Visual analogue scales (VAS) and categorical rating scales (CRS) were used to rate pain intensity, pain affect and temperature associated with the cold water pressor only. The VAS consisted of three 15 cm lines with endpoints designated as 'no pain' and 'extremely intense pain', 'not unpleasant' and 'extremely unpleasant', 'not cold' and 'extremely cold'. The CRS also provided measurements of intensity, affect and temperature using three 5 point scales in which $1 =$ 'no pain'/ 'not unpleasant'/ 'no sensation'; $2 =$ slightly [painful/ unpleasant/cold]; $3 =$ moderately [painful/ unpleasant/cold]; $4 =$ very [painful/ unpleasant/cold]; $5 =$ extremely [painful/ unpleasant/cold].

## 4. Results

Administration of each condition was accompanied by a loud vocal pain response by EQ. This was particularly marked in both aversive conditions, where there was evidence of an emotional dimension to his verbal and facial expression.

Inspection of the raw anticipatory and task activity SCL indicated little difference between the 'left' and 'right' somatosensory (innocuous and pin prick) and movement (passive and active) conditions for EQ. Therefore, the lateralised data were collapsed into a combined measure of anticipatory and task activity SCL for each condition. SCL recorded during both cold pressor trials were combined into a single mean anticipatory and task-activity measure for each participant (see Table 2).

### 4.1. Anticipatory and task activity SCL

Differences in anticipatory and task activity SCL from the pre-experimental baseline for the five conditions are presented in Fig. 2.

Table 1
Cognitive neuropsychological profile of patient EQ

| Neuropsychological test | EQ | Percentile/Range |
|---|---|---|
| *National Adult Reading Test* | 100 | Average (100–109) |
| *Wechsler Adult Intelligence Scales – Revised* | | |
| Verbal subtests | | |
| Information | 5 | 5th percentile |
| Digit span | 6 | 9th percentile |
| Vocabulary | 5 | 5th percentile |
| Arithmetic | 4 | 2nd percentile |
| Comprehension | 4 | 2nd percentile |
| Similarities | 3 | 1st percentile |
| Performance subtests | | |
| Picture completion | 5 | 5th percentile |
| Picture arrangement | 4 | 2nd percentile |
| Block design | 7 | 16th percentile |
| Object assembly | 3 | 1st percentile |
| Digit symbol | 2 | 1st percentile |
| Verbal IQ | 73 | Borderline (70–79) |
| Performance IQ | 77 | Borderline (70–79) |
| Full scale IQ | 75 | Borderline (70–79) |
| *Stroop* | | |
| Word | 71/112 | |
| Colour | 0/112 | $<$1st percentile |
| *Wisconsin Card Sorting Task* | | |
| Categories | 0 | $<$1st percentile |
| Percentage perseverative errors | 63% | 3rd percentile |
| *Test for Attentional Performance* | | |
| Phasic alertness | | |
| Median reaction time | 438.5 ms | $<$1st percentile |
| Visual attention Median reaction time | 952.0ms | $<$1st percentile |
| Divided attention Mean reaction time | 1003.0ms | $<$1st percentile |
| *Autobiographical Memory Interview* | | |
| Childhood | | |
| Personal semantic | 3/212 | Abnormal ($\geqslant$11) |
| Autobiographical incidents | 0/9 | Abnormal ($\geqslant$3) |
| Early adult life | | |
| Personal semantic | 4.5/21 | Abnormal ($\geqslant$14) |
| Autobiographical incidents | 0/9 | Abnormal ($\geqslant$3) |
| Recent life | | |
| Personal semantic | 0/21 | Abnormal ($\geqslant$17) |
| Autobiographical incidents | 0/9 | Abnormal ($\geqslant$5) |
| Total Personal semantic | 7.5/63 | Abnormal ($\geqslant$47) |
| Total Autobiographical incidents | 0/27 | Abnormal ($\geqslant$12) |
| *Rey Complex Figure* | | |
| Copy | 36/36 | No abnormalities detected |
| Immediate recall | 2/36 | $<$1st percentile |
| Delayed recall | 0/36 | $<$1st percentile |
| *Benton's Test of Unfamiliar Face Processing* | 41/54 | Normal (41–54) |
| *Unfamiliar/Famous Faces Sorting* | 50/50 | No abnormalities detected |
| *Recognition of Famous Faces* | 25/25 | No abnormalities detected |
| *Birmingham Object Recognition Battery* | | |
| Minimal feature view task | 24/25 | Normal range (18.5–25) |
| Foreshortened view | 23/25 | Normal range (16.7–25) |
| *Psycholinguistic Assessments of Language Processing* | | |
| Auditory synonym judgements | 54/60 | No abnormalities detected |

Although EQ displayed a heightened level of anticipatory autonomic arousal across *all* conditions, this only reached significance in the cold pressor condition (2 standard deviations above the normal control mean is the cut–off used to indicate impairment). Although the NC group displayed elevated SCL compared to base-

Table 2
Mean (SD) anticipatory and task activity skin conductance level ($\mu$mhos) for patient EQ and the normal control group across lateralised and combined conditions

| Condition | Participants | | | |
|---|---|---|---|---|
| | Patient EQ | | Normal Control group ($n = 4$) | |
| | Anticipatory | Task activity | Anticipatory | Task activity |
| *Left* | | | | |
| Passive | 5.62 | 5.88 | 5.84 (2.24) | 5.59 (2.15) |
| Active | 4.54 | 4.61 | 5.51 (1.72) | 5.76 (1.94) |
| Innocuous | 4.59 | 5.1 | 5.08 (1.54) | 5.13 (1.61) |
| Aversive [neurotip] | 4.61 | 4.96 | 5.53 (1.52) | 5.78 (1.69) |
| *Right* | | | | |
| Passive | 5.11 | 5.3 | 5.01 (1.41) | 4.86 (1.45) |
| Active | 5.35 | 5.36 | 4.89 (1.5) | 5.22 (1.72) |
| Innocuous | 5.22 | 5.99 | 5.0 (1.71) | 5.0 (1.79) |
| Aversive [neurotip] | 5.25 | 5.77 | 4.9 (1.63) | 5.6 (1.88) |
| *Combined* | | | | |
| Passive | 5.37 (0.43) | 5.59 (0.55) | 5.43 (1.79) | 5.22 (1.74) |
| Active | 4.95 (0.92) | 4.99 (0.82) | 5.2 (1.53) | 5.49 (1.72) |
| Innocuous | 4.91 (0.57) | 5.55 (0.60) | 5.03 (1.51) | 5.06 (1.58) |
| Aversive [neurotip] | 4.93 (0.85) | 5.37 (0.75) | 5.22 (1.50) | 5.32 (1.66) |
| Aversive [cold pressor] | 5.18 | 5.84 | 5.59 (1.734) | 5.3 (1.56) |

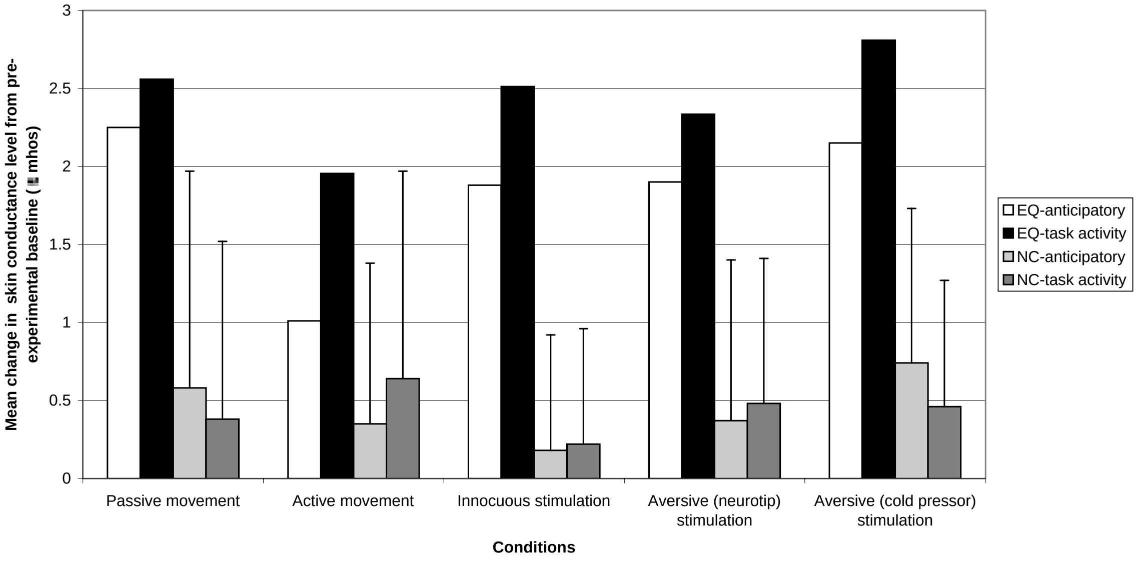

Pre-experimental baseline for EQ: 3.03 μmhos and for the normal control group: 4.85 (1 standard deviation=1.82) μmhos

Fig. 2. Differences in mean anticipatory and task activity skin conductance levels from pre-experimental baseline averaged across the movement and somatosensory stimulation conditions for patient EQ and normal control group (NC).

line, this was smaller relative to EQ.

The NC group displayed increased SCL from anticipatory levels during administration of active movement, innocuous and mild pin prick conditions, but decreases were evident during passive movement and the cold pressor test. In contrast, EQ's SCL was heightened during all activities, and in three conditions (innocuous stimulation, mild pin prick and cold pressor) it was more than two standard deviations above the NC group mean.

### 4.2. *Pain tolerance and qualitative evaluation of evoked sensations*

EQ was only able to keep his hand in the cold water for 11 seconds, which was significantly less time than

Table 3
Measurement of cold water tolerance (seconds), pain intensity, pain affect and temperature using visual analogue scales (15 cm lines) and categorical rating scales (5-points) are presented for cold water pressor for patient EQ and the normal control group

| | Participants | |
|---|---|---|
| | Patient EQ | Normal control group mean (1 standard deviation) |
| *Tolerance* (*sec*) | 11 | 240.63 (118.75) |
| Visual analogue scale | | |
| Pain intensity | 13.55 | 13.99 (4.44) |
| Pain affect | 14.1 | 14.79 (4.24) |
| Temperature | 14.9 | 13.46 (2.71) |
| Categorical rating scale | | |
| Pain intensity | 4 | 4 (0.65) |
| Pain affect | 4 | 4 (0.82) |
| Temperature | 4 | 4 (0.25) |

the NC group's mean of 241 seconds. In contrast, his ratings of pain intensity, pain affect and temperature did not differ from the NC group using either scale (see Table 3).

## 5. Discussion

It has been reported that frontal leucotomy [3], bilateral capsulotomy and cingulotomy [6,25] results in an elevated pain threshold and a reduction in the perceived unpleasantness of phasic heat stimuli in combination with a paradoxical decrease in tolerance for cold pain and exaggerated behavioural responses to trivial clinical procedures. Our findings are broadly consistent with these observations: EQ displayed normal ratings of pain intensity and pain affect [premorbid levels are not available] in the presence of a significantly reduced pain tolerance and an exaggerated behavioural response to somatosensory stimulation and movement.

EQ's data, alongside that reported by Davis et al. and Talbot et al. cannot be easily explained in terms of a dissociation between the sensory-discriminatory and affective-motivational components of the pain response, since ratings of both pain-intensity and pain-affect were within normal range. These findings have a number of important implications for the conceptualisation of the relationship between perception of affect and the sensory intensity of pain. Firstly, the strict dichotomy between the two components of the pain response may be an oversimplification. For instance, a number of psychophysical studies have reported a strong covariance between the ratings of intensity and unpleasantness for chronic and experimental pain (e.g. [7,22]). However, these findings must be considered with caution, given Ploner, Freund and Schnitzler's [21] report of a patient with a postcentral lesion who exhibited pain affect without pain sensation.

Secondly, Talbot et al. suggested that interruption of the thalamocortical pathways to cingulate and frontal cortices can significantly reduce both the unpleasantness and the sensory intensity of painful heat stimuli coupled with a decrease pain tolerance. This neuroanatomical account may also underlie EQ's performance profile. His MRI scan revealed abnormalities in subcortical white matter of the left Sylvian cortex; pronounced deficits in 'executive' functions, for example feedback to guide behaviour and response inhibition, were also evident. Executive functions are linked to prefrontal processing [16].

Thirdly, given that tolerance of pain generated by heat and cold dissociates from its perceived unpleasantness, this indicates that pain tolerance is not underpinned by the affective-motivational component of the pain response. In an attempt to explain these findings, Davis et al. proposed that under normal conditions the cingulate and prefrontal cortices are implicated in a mechanism which serves to suppress the subjective intensity of heat- and cold-pain. Consequently, the effect of cingulotomy and capsulotomy is to disinhibit appreciation of noxious heat and cold.

Fourthly, the presence of an exaggerated behavioural response to innocuous and mildly aversive somatosensory stimulation following frontal leucotomy, and also evident in EQ, was attributed to cognitive changes linked to increased apprehension rather than to an altered perception of somatosensory stimulation [3]. EQ's heightened skin conductance levels (SCL) across all tasks coupled with his preserved ability to accurately describe the form of innocuous stimulation taking place, appears to be consistent with this account.

EQ's arousal levels became pathologically elevated during administration of both innocuous and aversive somatosensory stimulation but not during either of the movement conditions. This finding may be explained in terms of modulation of somatosensory activity during voluntary movement. In a recent study, Whitford and Haggard [27] investigated the relationship between willed actions and the perceived intensity of somatic sensation. They found that voluntary actions produced reduced levels of conscious somatic sensation, and that this sensory suppression was blocked when a conditioning prepulse of transcranial magnetic stimulation was delivered over the supplementary motor area [SMA]. The authors concluded that the SMA may provide an efferent signal which is used by other brain areas to modulate somatosensory activity during voluntary action. Thus, EQ's ability to perform voluntary actions in the absence of an exaggerated behavioural response (e.g. to eat when hungry, to complete a crossword or puzzle), alongside the normal range SCL recorded during movement may, in part, be linked to sensory suppression.

Finally, an important limitation of the current study is worth mentioning. Our design did not account for individual differences in personality or mood between EQ and the NC group. Findings from a recent functional imaging study suggest that the context of presentation, personal experience relating to the 'emotional' content, the mood state and affective style/personality can influence brain reactivity to emotional stimuli [25].
